# Investigation of physio-mechanical, antioxidant and antimicrobial properties of starch–zinc oxide nanoparticles active films reinforced with *Ferula gummosa* Boiss essential oil

**DOI:** 10.1038/s41598-024-56062-w

**Published:** 2024-03-09

**Authors:** Hamid Sarhadi, Fatemeh Shahdadi, Ali Salehi Sardoei, Mehrnaz Hatami, Mansour Ghorbanpour

**Affiliations:** 1 Department of Food Science, Bam Branch, Islamic Azad University, Bam, Iran; 2 Department of Food Science and Technology, Faculty of Agriculture, University of Jiroft, Jiroft, Iran; 3 Crop and Horticultural Science Research Department, South Kerman Agricultural and Natural Resources Research and Education Center, AREEO, Jiroft, Iran; 4 Department of Medicinal Plants, Faculty of Agriculture and Natural Resources, Arak University, Arak, 38156-8-8349 Iran

**Keywords:** Active film, Antimicrobial activity, ZnO NPs, *Ferula gummosa* oil, Mechanical properties, Salmon fillets, Biochemistry, Plant sciences

## Abstract

The production of surface compounds coated with active substances has gained significant attention in recent years. This study investigated the physical, mechanical, antioxidant, and antimicrobial properties of a composite made of starch and zinc oxide nanoparticles (ZnO NPs) containing various concentrations of *Ferula gummosa* essential oil (0.5%, 1%, and 1.5%). The addition of ZnO NPs improved the thickness, mechanical and microbial properties, and reduced the water vapor permeability of the starch active film. The addition of *F. gummosa* essential oil to the starch nanocomposite decreased the water vapor permeability from 6.25 to 5.63 g mm^−2^ d^−1^ kPa^−1^, but this decrease was significant only at the concentration of 1.5% of essential oils (*p* < 0.05). Adding 1.5% of *F. gummosa* essential oil to starch nanocomposite led to a decrease in Tensile Strength value, while an increase in Elongation at Break values was observed. The results of the antimicrobial activity of the nanocomposite revealed that the pure starch film did not show any lack of growth zone. The addition of ZnO NPs to the starch matrix resulted in antimicrobial activity on both studied bacteria (*Staphylococcus aureus* and *Escherichia coli*). The highest antimicrobial activity was observed in the starch/ZnO NPs film containing 1.5% essential oil with an inhibition zone of 340 mm^2^ on *S. aureus*. Antioxidant activity increased significantly with increasing concentration of *F. gummosa* essential oil (*P* < 0.05). The film containing 1.5% essential oil had the highest (50.5%) antioxidant activity. Coating also improved the chemical characteristics of fish fillet. In conclusion, the starch nanocomposite containing ZnO NPs and *F. gummosa* essential oil has the potential to be used in the aquatic packaging industry.

## Introduction

The use of synthetic polymers for food packaging has been of interest to manufacturers for many years. These polymers provide protection to food against adverse environmental conditions such as microbes, mechanical stress, and chemicals. Polyethylene, polypropylene, and polyvinyl chloride are examples of synthetic polymers that are typically non-polar and derived from non-renewable oil sources^1^. A solution to this problem is the use of renewable polymers, which can offer benefits such as biodegradability, low toxicity, low manufacturing cost, and low waste disposal cost^2^. Starch is an edible polymer that can be used to make biodegradable films due to its suitable properties for film formation. However, biodegradable polymers have limitations such as inappropriate permeability to gases, unfavorable mechanical properties, and low thermal degradation temperature, which restrict their industrial use^3^. Recently, nanotechnology has been used to address these limitations. Nanotechnology involves controlling materials in the approximate dimensions of 1–100 nm^4^. The high surface-to-volume ratio of nanofillers makes the interfacial forces between nanofillers and biopolymers stronger, resulting in the formation of nanocomposites^5^. Among the different nanofillers, ZnO NPs is a polar inorganic crystalline compound with a hexagonal quartz structure^6^. The physical and chemical properties of ZnO NPs depend on the size and shape of its nanostructure, which can be spherical, nanorod, needle-shaped, or flower-like, depending on the preparation method.

Plant essential oils are a group of aromatic and secondary metabolites found in plants that possess antimicrobial and antioxidant properties. They are a complex mixture of natural compounds such as terpenoids, terpenes, phenolic acids, and other aliphatic and aromatic compounds^7^. One such plant is *F. gummosa*, which is a member of the Umbelliferae family and has an essential oil with antidiabetic, antimicrobial, and antioxidant properties^1^. Although plant essential oils have favorable effects, adding them directly to food can cause problems such as toxicity, strong odors, and changes in organoleptic characteristics. A solution to this problem is adding essential oils to packaging films as active packaging, which protects the natural compounds and allows for a controlled release of essential oils into food^8^. Heydari Majd et al.^6^ conducted research on the effect of thyme, peppermint, and ZnO NPs essential oils on the properties of polylactic acid active films. The results showed that these active films increased the shelf life of fish meat from 8 to 16 days due to their strong antimicrobial and antioxidant properties. Javidi et al.^9^ produced a biodegradable polylactic acid (PLA) film containing *Origanum majorana* essential oil. The antimicrobial test of the film revealed that *S. aureus* was the most sensitive bacterium to the prepared nanocomposite, while *E. coli* was the most resistant.

The purpose of this research was to examine the physical and mechanical features, as well as the antibacterial and antioxidant effects, of active films composed of starch, ZnO NPs, and varying concentrations of essential oils. Additionally, the study aimed to assess the effectiveness of a starch/ZnO NPs composite that contains *F. gummosa* essential oil in prolonging the shelf life of rainbow salmon fillets.

## Materials and methods

### Chemicals

Starch (moisture 12%) was prepared from Chemi-Bazar company. Nano zinc oxide (purity grade > 99%, particle size 10–30 nm) was purchased from Pisgaman Nano Materials Iranian Company. Folin–Ciocalteu’s reagent, 2,2-diphenylpicrylhydrazyl (DPPH), gallic acid and sodium carbonate were obtained from Merck company (Germany). Mueller Hinton agar were purchased from Sigma company (USA).

### Preparation of starch nanocomposite containing ZnO NPs and *F. gummosa* essential oil

For this purpose, the casting method was used. At first, to prepare a 4% starch aqueous solution, a temperature of 90 °C was used for 45 Min to complete the starch gelatinization stage. Then, glycerol plasticizer (45% w/w) was added to the solution in order to prevent the brittleness of the films. Next, amounts of 0.5, 1 and 1.5% of ZnO NPs were added to the solution after being homogenized by ultrasound for 5 Min. *F. gummosa* essential oil was added to the starch solution at 0.5%, 1% and 1.5% concentrations. Finally, this solution was homogenized by an Ultratrax homogenizer (at a speed of 10,000 RPM for 5 Min). To dry the nanocomposite solution, it was poured into a glass plate with dimensions of 15 × 10 cm^2^ and a thickness of 1.5 mm, and it was dried overnight in laboratory conditions. Then the dried films were separated from the surface of the plate and kept in a desiccator^6^.

### Physicochemical tests

#### Thickness

A digital micrometer with an accuracy of 0.0001 mm was used to measure the thickness of the films.

#### The water vapor permeability of films (WVP)

The permeability of starch nanocomposites to water vapor was determined based on the modified method of E96 approved by ASTM. After reaching the moisture balance, the films were fixed by melted paraffin on the surface of the measuring cells and then placed in a desiccator containing silica gel. Water at a temperature of 25 °C creates 100% humidity. The difference in humidity on the two sides of the film at a temperature of 25 °C creates a difference in heating pressure equal to 2.337 × 10^3^ Pa. The test cells were weighed at zero time and 8 h after the start of the test using a digital scale and their weight changes were recorded. The water vapor permeability of starch nanocomposites was obtained according to the Eq. (1):

1 $$ \begin{aligned} {\text{WVP}} & = {\text{Weight Loss}}\; \times \;{\text{Film thickness}}/{\text{The exposed surface of the film}}\; \times \;{\text{Time }} \\ & \quad \times \;{\text{The difference in vapor pressure on both sides of the film}} \\ \end{aligned} $$

### Mechanical properties

The most common tests to evaluate the mechanical properties of a nanocomposite are the Tensile strength and Elongation at break. These characteristics were calculated according to the ASTM-D882-02 standard method and using a Texture Analyzer (model Plus-TA, England). First, the film was cut to the dimensions of 1 × 10 cm^2^ and its thickness was calculated at 10 random points. The samples were conditioned for 2 days in a desiccator containing calcium nitrate solution with 55% humidity. Next, in order to evaluate the mechanical characteristics of the samples, a texture measuring device was placed between the two jaws with an initial distance of 100 mm and a jaw movement speed of 12.5 mL/Min. Mechanical properties were calculated according to the Eqs. (2) and (3):

2 $$\mathrm{Tensile\; strength}=\frac{{{\text{F}}}_{{\text{max}}}}{{{\text{A}}}_{{\text{min}}}}$$

In this equation: F_max_ is the maximum force applied to the film in Newtons and A_min_ is the smallest initial cross-sectional area of the sample in m^2^.

3 $$\mathrm{Elongation\; at\; break\%}=\frac{{{\text{E}}}_{{\text{b}}}}{{{\text{D}}}_{{\text{i}}}}$$

In this equation: E_b_ is the amount of elongation until the moment of rupture and D_i_ is the initial distance between the two jaws^10^.

### Determination of antimicrobial activity

**Figure 1:**
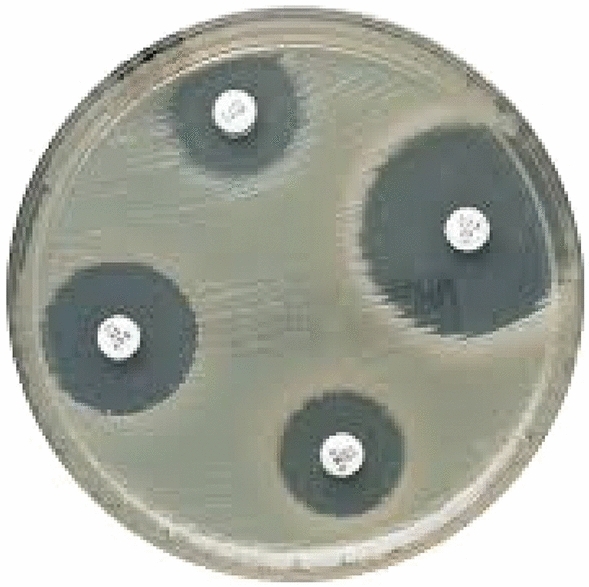
The diameter of inhibition zone area.

In order to investigate the antimicrobial activity of nanocomposite samples against *E. coli* and *S. aureus*, the disk diffusion method was used. For this purpose, pieces of film with a diameter of 10 mm were prepared and sterilized. Before placing the sterile discs on the culture medium, the surface culture process was performed with 100 µL of liquid culture medium containing approximately 10^8^ CFU/mL of each of the mentioned pathogenic bacteria. In the following, the discs were placed on the Mollier Hinton agar culture medium and then kept at 37 °C for 24 h. Finally, the area of inhibition zone (halo of non-growth) was measured using a digital caliper (Fig. 1) in mm^11^.

### Total phenolic content

The total phenolic content was determined using Folin–Ciocalteu’s reagent method according to the Moradi et al.^12^. For this purpose, first, 20 mg of nanocomposite sample was dissolved in 5 mL of distilled water, and then 0.1 mL of the obtained solution was mixed with 6 mL of distilled water and 0.5 mL of Folin–Ciocalteu’s reagent. The obtained solution was kept at room temperature for about 8 Min and then 1.5 mL of sodium carbonate solution (2%) and 0.9 mL of distilled water were added to make the final volume 10 mL. The resulting solution was kept in the dark at room temperature for 1 h. Finally, the absorbance of the solution was measured by a spectrophotometer (Shimadzu UV–VIS 1601, Japan) at 765 nM. The calibration curve was drawn using gallic acid as the standard of phenolic compounds. Total phenolic content is calculated as mm gallic acid per gdry matter according to the Eq. (4):

4 $${\text{T}}=\frac{{\text{C}}\cdot{\text{V}}}{{{\rm M}}}$$

where in this equation: T: total phenol content (mg/g of dry matter), C: concentration of gallic acid calculated from the calibration curve (mg/ml), V: volume of nanocomposite extract (mL) and M: weight of dry nanocomposite (g).

### Evaluation of antioxidant properties of nanocomposite using free radical scavenging

The antioxidant activity of the films was performed by examining the inhibition of DPPH free radicals according to the method of Salehi Sardoei and Shahdadi^13^. Therefore, at first, 20 g of the nanocomposite was dissolved in 5 mL of distilled water, then 0.1 mL of this solution was mixed with about 3.9 mL of DPPH methanolic solution (0.1 M). The reaction mixture was stirred and placed in total darkness at room temperature for 2 h. Finally, the absorbance of the resulting solution was measured at 517 nM using a spectrophotometer. The percentage of free radical inhibitory activity was calculated according to the Eq. (5):

5 $$\mathrm{DPPH\; Scavenging\; activity\; }\left(\mathrm{\%}\right)=\left(\frac{{{\text{A}}}_{{\text{blank}}}-{{\text{A}}}_{{\text{sample}}}}{{{\text{A}}}_{{\text{blank}}}}\right) \times 100$$

In this equation, A_blank_ is the absorbance value of methanolic DPPH solution without adding nanocomposite and A_sample_ is the absorbance value of the tested sample.

### Preparation and coating of salmon fillets

Salmon weighing approximately (250 ± 25 g) was purchased from the coast of Chabahar Sea in Sistan and Baluchistan province and was immediately transported to the food laboratory in an ice container. After draining the guts and entrails as well as separating the head, tail and skin, the fish were washed well with water and then placed on a net to drain their water. The fishes were divided into the following groups:

1. Control: fish samples without packaging.
2. Fish sample packed in film containing nanoparticles without essential oil
3. Fish sample packed in film containing nanoparticles and 0.5% essential oil
4. Fish sample packed in film containing nanoparticles and 1% essential oil
5. Fish sample packed in film containing nanoparticles and 1.5% essential oil

At the end, the packed fillet samples were kept at 4 °C for 16 days and subjected to microbial and chemical tests every 4 days.

### Microbial analysis of salmon fillet

Briefly, 20 g of fish fillet was weighed under sterile conditions. Next, fish fillets were mixed with 225 mL of peptone water solution (0.1%) and homogenized in a mixer at 400 RPM for 2 Min. Then the obtained solution was diluted until a dilution of one tenth was obtained. For the purpose of microbial investigation, the following culture media were used: In order to investigate the total microbial count, the Plate Count Agar (PCA) medium was used at 30 °C for 48 h^14^. In order to count lactic acid bacteria, de Man, Rogosa, and Sharpe agar (MRS) culture medium at 25 °C for 5 days under anaerobic conditions was used^15^. In order to count *Pseudomonas* bacteria, one-tenth dilution of the initial solution was placed on Pseudomonas Agar Base (Oxoid) supplemented with CFC (Cetrimide Fucidine Cephalosporine, Oxoid code CM 559, supplemented with SR 103, Oxoid, Basingstoke) and it was kept in a at 20 °C for 48 h^6^. All tests were performed in three repetitions. Microbial counts were expressed as log CFU/g.

### Chemical analysis of fish fillet

#### pH measurement

In order to measure the pH, 4 g of sample was homogenized with 10 mL of distilled water. Then, using a pH meter, the pH of fish fillets was measured and recorded at room temperature.

#### Measurement of total volatilebase nitrogen (TVB-N)

The TVB-N test was performed using the titration method as well as steam distillation^6^. The amount of total volatile nitrogen was calculated in mg per 100 g of fish fillet.

#### Thiobarbituric acid (TBA) index

Malonaldehyde is one of the most important products resulting from the decomposition of hydroperoxide in the oxidation of lipids. Today, the most common method of evaluating the amount of malonaldehyde in food samples is the TBA test. The basis of this test is based on measuring the amount of pink color resulting from the reaction between malonaldehyde and two TBA molecules by spectroscopic method^6^. For this purpose, 6 g fish fillet sample was homogenized with 15 mL of distilled water. Then 1 mL of the resulting mixture was completely mixed with 2 mL of the TCA/TBA mixture (TBA 15 mM and TCA 15%) and placed in a water bath at 90–100 °C for 30 Min to create a pink color. Then it was placed in cold water for 10 Min and centrifuged for 20 Min. The absorbance of the supernatant was read at 531 nm. The control solution containing one mler of distilled water and 2 mL of TCA/TBA solution was used.

### Statistical analysis

In different parts of the research, all tests were performed at least three times. Analysis of variance (ANOVA) was performed using SPSS statistical software in the form of completely randomized design. Means were compared at the 5% error level with Duncan’s multi-range test.

## Results and discussion

**Figure 2:**
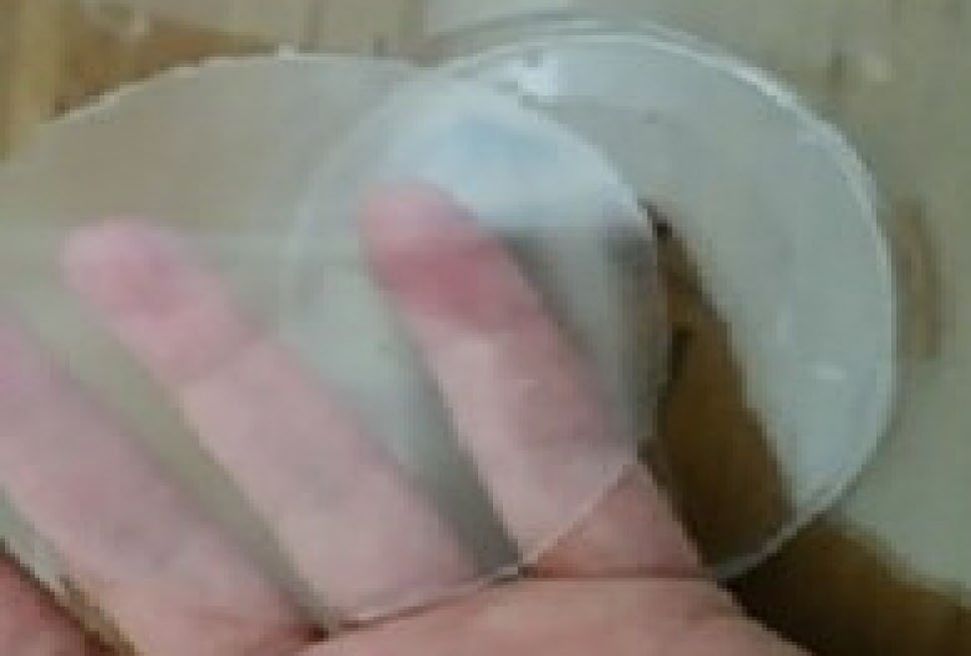
Starch film.

The casting method used in this research led to the production of starch film with proper flexibility and no breakage, so that the produced film was easily separated from the surface of the plate. Also, as shown in Fig. 2, the produced film had a smooth and transparent surface.

### Physical tests

#### Thickness of starch nanocomposite

The thickness of a film is directly related to the microstructure and orientation of molecules in that film^16^. Also, the thickness of a film is linearly related to mechanical properties and moisture permeability. The thickness of starch nanocomposite containing different concentrations of ZnO NPs and *F. gummosa* essential oil is shown in Table 1. The simultaneous addition of *F. gummosa* essential oil along with ZnO NPs to the starch film increased the thickness, which ranged from 0.090 to 0.102 mm. The nanocomposite containing 1.5% ZnO NPs and 1.5% *F. gummosa* essential oil had the highest thickness (0.102 mm) (Table 1). Adding ZnO NPs and basil leaves essential oil to the composite film based on fish protein isolate and fish skin gelatin also increased the thickness^17^. Depending on the nature of the nanoparticle used and the type of polymer in different studies, the addition of nanoparticles has increased the thickness^2^ and in some studies it has decreased the thickness^18^ of the produced film. Therefore, nanoparticles have different effects on the thickness of nanocomposite. This increase in thickness is probably due to the presence of essential oil and ZnO NPs in the middle of the starch polymer network and the increase in the solid content of the produced film^17^.

**Table 1:** Physical properties of nanocomposite films and nanocomposite combination with ZnO NPs and *Ferula gummosa* essential oil.

Film	Thickness (mm)	Water vapor permeability (g mm^−2^ d^−1^ kPa^−1^)
Starch	0.09 ± 0.003c*	7.88 ± 0.275a
S + 0.5%ZnO NPs	0.09 ± 0.002c	7.56 ± 0.132ab
S + 1%ZnO NPs	0.092 ± 0.001bc	7.01 ± 0.050b
S + 1.5%ZnO NPs	0.097 ± 0.003abc	6.35 ± 0.152c
S + 1.5%ZnO NPs + 0.5%EO	0.099 ± 0.004ab	6.25 ± 0.306c
S + 1.5%ZnO NPs + 1%EO	0.1 ± 0.002ab	6.05 ± 0.176cd
S + 1.5%ZnO NPs + 1.5%EO	0.102 ± 0.002a	5.63 ± 0.229d

*Different letters in a column indicate a significant difference between the means (*p* < 0.05).

#### Water vapor permeability (WVP)

Moisture is one of the influential factors in the reactions leading to spoilage and the growth of microorganisms in food products, and therefore, the water vapor permeability of a polymer should be as low as possible to increase the shelf life of food^19^. The permeability to water vapor in the pure starch film was 7.88 g mm^−2^ d^−1^ kPa^−1^ (Table 1). This value is comparable to the WVP obtained for pure starch (7.50 g mm^−2^ d^−1^ kPa^−1^) in the study of Ghasemlou et al.^20^. As can be seen in Table 1, the addition of ZnO NPs decreased the water vapor permeability of the starch film. In similar studies, Arfat et al.^17^ reported that adding ZnO NPs reduced the water vapor permeability of fish protein isolate film. These researchers stated that the reason for this is the increase of meandering paths along the length of the polymer due to the addition of nanoparticles, which create a barrier against the passage of water molecules.

Considering that the resistance to water vapor of the starch film containing ZnO NPs is still not comparable to many common plastic films, therefore, to solve this problem, adding different hydrophobic compounds such as plant essential oils is a possible solution. Therefore, in the following, the effects of adding *F. gummosa* essential oil on the water vapor permeability of the starch film containing ZnO NPs were investigated. The addition of *F. gummosa* essential oil to starch nanocomposite decreased the permeability to water vapor from 6.25 to 5.63 g mm^−2^ d^−1^ kPa^−1^, but this decrease was significant only in the concentration of 1.5% of *F. gummosa* essential oils (*p* < 0.05). The decrease in permeability to water vapor of the film due to the addition of essential oil is related to the hydrophobic nature of the essential oil, which affects the hydrophilic/hydrophobic properties of the film and thus leads to a decrease in the affinity of the starch film to water and a decrease in permeability to water vapor^21^. Similar results with this study by Salarbashi et al.^11,13^ for film based on soybean soluble polysaccharide due to the addition of thyme and oregano essential oils, and also by Shojaee Aliabadi et al.^22^ for carrageenan based film due to the addition of *Satureja hortensis* essential oil was observed.

### Mechanical properties

A film with appropriate characteristics must be sufficiently resistant to environmental pressures during transportation, processing, and storage to maintain the integrity of food ingredients^23^. Tensile strength (TS) and elongation to breaking point (EB) are among the main properties to investigate the mechanical properties of a nanocomposite in food applications. One of the disadvantages of starch film is its brittle behavior. On the other hand, adding essential oil to the nanocomposite produces a film with a heterogeneous matrix. *F. gummosa* essential oil reduces the overall cohesion of starch polymer network forces and therefore reduces the tensile strength of the emulsified starch film. Therefore, the addition of ZnO NPs has been investigated considering the behavior of essential oil on the mechanical properties of the starch film. The pure starch film has an elongation to breaking point of about 28%, which is comparable to the values reported for pure starch in previous studies (29%)^24^. According to Table 2, the values of TS and EB of nanocomposite reached 16 and 27% Mpa from 14 and 28, respectively, by adding 1.5% ZnO NPs. In a similar study, the addition of ZnO NPs to starch foam increased the amount of TS and decreased the amount of EB^21^. The increase in the tensile strength of the starch film can be due to the filling of the empty spaces of the starch polymer by ZnO NPs and the creation of new strong bonds in the polymer^25^.

**Table 2:** The mechanical properties of starch nanocomposite.

Film	TS (MPa)	EB (%)
Starch	14.0 ± 6.20c*	28.0 ± 2.25d
S + 0.5%ZnO NPs	14.0 ± 7.16c	28.0 ± 3.07d
S + 1%ZnO NPs	15.0 ± 1.17b	27.0 ± 2.60e
S + 1.5%ZnO NPs	16.0 ± 1.15a	27.0 ± 1.10e
S + 1.5%ZnO NPs + 0.5%EO	15.0 ± 2.21b	29.0 ± 1.30c
S + 1.5%ZnO NPs + 1%EO	13.0 ± 5.10d	31.0 ± 2.26b
S + 1.5%ZnO NPs + 1.5%EO	10.0 ± 1.12e	33.0 ± 2.07a

*Different letters in a column indicate a significant difference between the means (*p* < 0.05).

The results showed that the addition of *F. gummosa* essential oil to the starch nanocomposite caused a decrease in the amount of TS. For example, adding 1.5% of *F. gummosa* essential oil caused a 62% decrease in TS value. This effect can be due to the relative replacement of the strong polymer–polymer network forces in the starch by the weak complex structure formed between the lipids and the starch chain in the film network in the presence of Barijah essential oil, which causes the overall cohesion of the polymer network forces to become weaker and therefore the tensile strength. reduce the emulsified film^26^. According to Table 2, up to 1.23 times increase in EB values was obtained by adding 1.5% of *F. gummosa* essential oil to starch. The results of this study are consistent with the results for polylactic acid containing active ingredients such as cinnamic aldehyde or thymol^27^.

By examining the results related to the physical and mechanical properties of starch nanocomposite, 1.5% concentration of ZnO NPs was chosen as the best concentration, and further tests were performed only on this concentration.

### Antimicrobial activity of films

The results of antimicrobial activity of starch nanocomposite containing 1.5% of ZnO NPs and different concentrations of *F. gummosa* essential oil against *S. aureus* and *E. coli* are shown in Table 3. The results of the antimicrobial activity of the nanocomposite showed that the pure starch film did not show any lack of growth zone. The addition of ZnO NPs to the starch matrix caused antimicrobial activity on both studied bacteria. The antimicrobial mechanisms of ZnO NPs are probably due to the release of Zn^2+^ ions, the production of OH^▪^ and O_2_^▪^ radicals, as well as the penetration of the nanoparticle itself into the cell, which causes the destruction of the bacterial cell membrane, interaction with the compounds in the cytoplasm, and finally the death of the bacteria^28^. Similar results were reported by Mirjalali et al.^29^ regarding the antimicrobial activity of ZnO NPs on pathogenic bacteria.

**Table 3:** The antimicrobial properties of starch nanocomposite.

Film	Zone of inhibition (mm^2^)	
	*S. aureus*	*E. coli*
Starch	NDe*	NDe
S + 1.5%ZnO	30.15 ± 3.50d	19.14 ± 2.11e
S + 1.5%ZnO + 0.5%EO	80.20 ± 3.50c	55.10 ± 1.70d
S + 1.5%ZnO + 1%EO	220.55 ± 3.55b	150.50 ± 3.40b
S + 1.5%ZnO + 1.5%EO	340.70 ± 3.22a	260.40 ± 5.17a

*Different letters in a column indicate a significant difference between the means (*p* < 0.05), ND: Not Detected.

Addition of *F. gummosa* essential oil with synergistic effect improved the antimicrobial activity of starch nanocomposite containing ZnO NPs (Table 3). The reason for this antimicrobial activity is due to the penetration of *F. gummosa* essential oil through the agar gel and finally creating a specific zone around the production discs. Increasing the concentration of *F. gummosa* essential oil improved the antimicrobial activity. The highest antimicrobial activity was observed in the starch/ZnO NPs film containing 1.5% *F. gummosa* essential oil with an inhibition zone of 340 mm on *S. aureus*. Also, the results showed that the inhibition rate of *E. coli* was lower than that of *S. aureus*. This means that Gram-negative bacteria showed more resistance to *F. gummosa* essential oil. *E. coli* is a Gram-negative bacterium, and having an extra external membrane around its cell wall can prevent the release of hydrophobic compounds across its liposaccharide membrane^30^. Similar results were reported by Heydari Majd et al.^6^, in which polylactic acid-based film containing ZnO NPs and each of peppermint and thyme essential oils inhibited the growth of gram-positive bacteria more than gram-negative bacteria. The antimicrobial activity of *F. gummosa* essential oil can be due to the presence of phenolic compounds in it such as alpha and beta pinene^1^. The antimicrobial mechanisms of these compounds can be due to disruption of membrane permeability and intracellular content of bacteria and interference in the pathway of synthesis of respiratory enzymes in mitochondria^31^.

### Total phenolic compounds

**Figure 3:**
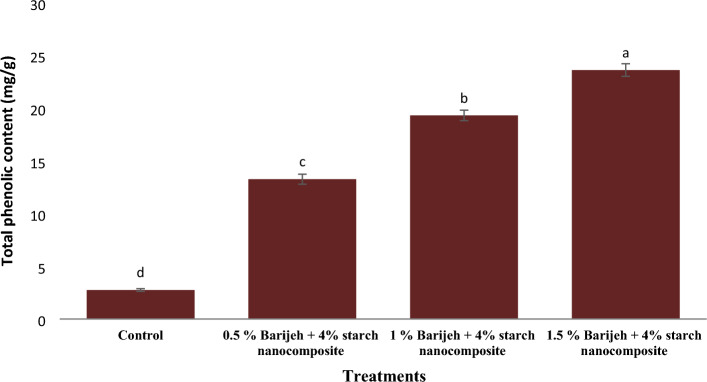
Phenol content of starch nanocomposite containing different concentrations of *Ferula gummosa* essential oil. Non-identical Latin letters indicate significant difference (*p* < 0.05) between means.

The effective ingredients in essential oils are generally phenolic compounds that have the ability to inhibit malondialdehyde, which is one of the important products of fat oxidation^6^. The total phenolic content results showed that the pure starch (control sample) showed a very low total phenolic content (2.75 mg/g) (Fig. 3). It should be noted that this amount of total phenolic compounds reported is not related to the phenolic content of pure starch, but it is related to non-phenolic compounds that are present in the starch film due to their reducing properties, and these compounds cause creates colored compounds^32^. Similar results were observed by Heydari Majd et al.^6^ for the phenol content of pure polylactic acid film. The amount of total phenolic compounds in the starch nanocomposite showed a significant increase with the increase in the concentration of *F. gummosa* essential oil, so that the highest amount of total phenol (23.7 mg Galic acid/g sample) was observed in the nanocomposite containing 1.5% *F. gummosa* essential oil. Similar results were observed by adding thyme and peppermint essential oils to the active carbohydrate film extracted from soy protein^2^. One of the most important phenolic compounds in *F. gummosa* essential oil can be mentioned alpha and beta pinene.

### Free radical scavenging activity

**Figure 4:**
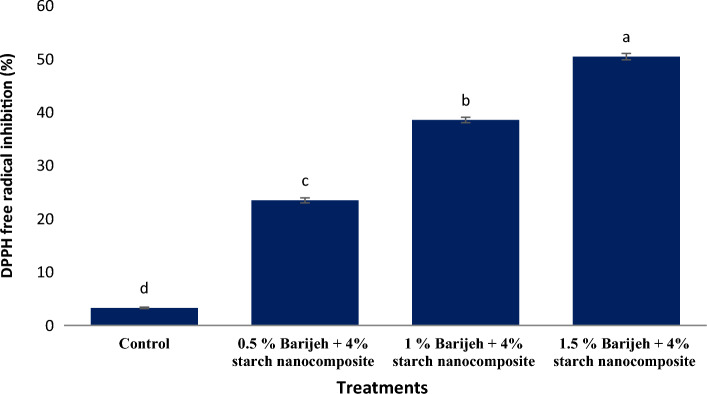
Antioxidant activity of starch film containing different concentrations of *Ferula gummosa* essential oils. Non-identical Latin letters indicate significant differences (*p* < 0.05) between the means.

The free radical scavenging activity of starch nanocomposite was evaluated according to the inhibition of DPPH radicals. Figure 4 shows the amount of antioxidant activity of starch nanocomposite. Antioxidant activity increased significantly (*P* < 0.05) with increasing concentration of *F. gummosa* essential oil. The film containing 1.5% *F. gummosa* essential oil had the highest (50.5%) antioxidant activity. The review of sources shows that essential oils and plant extracts are capable of inhibiting free radicals due to their effective compounds including phenolic compounds. For example, Zein nanofibers showed strong antioxidant properties due to the addition of *F. gummosa* essential oil^6^.

In the study of Mondal et al.^33^ the control film exhibited a moderate scavenging activity. However, a remarkable improvement in DPPH antioxidant activity was observed in the developed chitosan/crude algae ethanolic extract edible films. The DPPH radical-scavenging activity of the films increased with the increasing concentration of crude algae ethanolic extract. The rise in antioxidant activity could be attributed to the presence of phenolic compounds and a small fraction of –CH_2_–groups in extract^33,34^.

### Chemical analysis of fish fillet

#### pH

Generally, the pH of fresh fish meat is almost neutral^35^. The effect of starch nanocomposite containing ZnO NPs and different concentrations of *F. gummosa* Boiss essential oil on the pH changes of salmon fillets during 16 days of storage at refrigerator temperature is shown in Table 4.

**Table 4:** pH values of salmon fillet coated by starch nanocomposite.

Film	First day	4th day	8th day	12th day	16th day
Starch	6.27 ± 0.02bc*	6.18 ± 0.01cd	6.30 ± 0.03bc	6.43 ± 0.04ab	6.60 ± 0.01a
S + 1.5%ZnO	6.27 ± 0.01bc	6.16 ± 0.02cd	6.25 ± 0.01c	6.31 ± 0.03bc	6.46 ± 0.01ab
S + 1.5%ZnO + 0.5%EO	6.27 ± 0.02bc	6.14 ± 0.01d	6.24 ± 0.02c	6.29 ± 0.02bc	6.44 ± 0.02ab
S + 1.5%ZnO + 1%EO	6.27 ± 0.01bc	6.11 ± 0.04d	6.21 ± 0.01cd	6.27 ± 0.02bc	6.42 ± 0.03ab
S + 1.5%ZnO + 1.5%EO	6.27 ± 0.03bc	6.10 ± 0.02d	6.17 ± 0.03cd	6.23 ± 0.01c	6.40 ± 0.03b

*In each row and column, the numbers with different letters have statistically significant differences (*p* < 0.05).

The initial pH (first day) of the uncoated sample (control), coated with starch containing 1.5% ZnO NPs and three concentrations of *F. gummosa* essential oil was measured as 6.27 (Table 4), which is similar The results of Jebeli Javan et al.^36^ who investigated the effect of *Scrophularia striata* aqueous extract on rainbow trout fillets. The pH of all samples showed a decreasing trend and then an increasing trend during the first 4 days. The initial decrease in the pH values of all samples is due to the breakdown of glycogen in the muscle of the fish fillet after catching, although some studies consider the dissolution of carbon dioxide in the aqueous phase of the fish muscle and finally the formation of carbonic acid as the reason for the decrease in pH^36^. Also, the increase in pH during the storage period is related to the production of nitrogen products such as ammonia, trimethylamine and other biogenic amines produced by the bacteria^37^. A difference was observed between the control and the samples coated with starch nanocomposite and coated with *F. gummosa* essential oil until the end of the storage period, and this difference was completely significant at the end of the storage time (*P* < 0.05). The lower pH value in the samples containing the coating and *F. gummosa* essential oil compared to the control sample is due to the effect of the film and *F. gummosa* essential oil on inhibiting the activity of internal enzymes as well as the inhibitory effect on the activity of pathogenic bacteria and therefore increasing the shelf life of these fillets^38^. Channamarana et al.^39^ in the study of shelf life of salmon by chitosan film and thyme essential oil reported that the lowest increase in pH was related to the samples coated by chitosan with thyme essential oil, chitosan coating and control, respectively. Also, Bazargani Gilani et al.^40^ reported that chicken breast coated with chitosan film containing pomegranate juice and thyme essential oil caused a significant decrease in pH at the end of the storage period compared to the control.

#### Thiobarbituric acid index (TBA)

**Figure 5:**
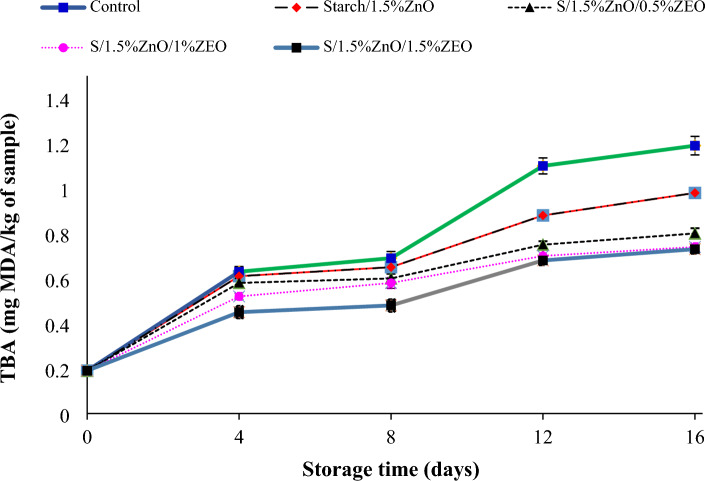
Changes in tebiobarbiuric acid (TBA) in salmon fillet during storage in the refrigerator.

The effect of starch nanocomposite containing ZnO NPs and *F. gummosa* essential oil on the amount of thiobarbituric acid in fish fillets during 16 days of storage at refrigerated temperature is shown in Fig. 5. The initial TBA of the samples was about 0.19. From the fourth day onwards, an increasing trend was observed in the amount of TBA index during the maintenance period. Also, from the 4th day to the 16th day, there was a significant difference (*P* < 0.05) between the samples, in which the highest amount of TBA was observed in the control, starch nanocomposite, and *F. gummosa* essential oil nanocomposite, respectively. According to the standard, the acceptable amount for the TBA index is 1 mg of malonaldehyde/kg of fish meat^41^. The value of this index on the last day of storage for the control, starch nanocomposite and 1.5% concentration of *F. gummosa* essential oil with nanocomposite was 1.19, 0.98 and 0.73 mg of malonaldehyde/kg of fish meat respectively, which is lower than acceptable standard limit. In general, the lower amount of TBA index in the samples containing nanocomposite and *F. gummosa* essential oil can be attributed to the antioxidant activity of *F. gummosa* essential oil and their ability to break the chain of free radicals and chelate metal cations or donate hydrogen atoms^41^.

Nami et al.^42^ investigated the anti-oxidative effects of sodium alginate edible film containing vitamin C on the quality of rainbow salmon fillets and reported that with the passage of time, TBA values increased significantly (*P* < 0.05) in all treatments and the lowest amount of TBA was related to the coating containing vitamin C. Kostaki et al.^43^ reported that the small amount of TBA index in the samples coated with sodium alginate film is due to the barrier of the film from oxygen penetration. But the low TBA index for the sodium alginate sample containing vitamin C was due to the antioxidant effects of this vitamin.

Noshad et al.^44^ reported that the edible coating of edible coating based on *Plantago major* seed mucilage and Citrus limon essential oil reduced the progression of lipid oxidation in buffalo meat during storage.

In a study by Alizadeh Behbahani et al.^45^ addition of chicory essential oil into *Lepidium perfoliatum* seed mucilage edible coating decreased the TBA progression, and all the coated beef slices had lower TBA levels than the acceptable limit. This study showed that the use of chicory essential oil-rich edible coating was effective in delaying the oxidation of beef during 7 days of refrigeration display, probably due to the antioxidant activity of the oil and the ability of the edible coating to minimize the contact with oxygen and light^46^.

#### TVB-N results

**Figure 6:**
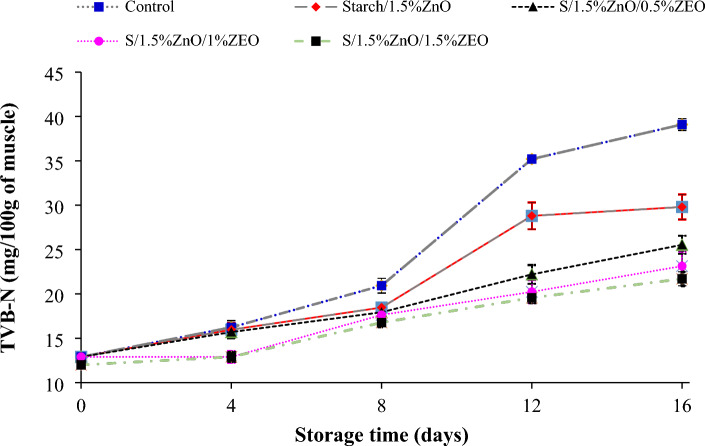
Changes in the amount of volatile nitrogen compounds (TVB-N) in salmon fillet during storage in the refrigerator.

The effect of starch nanocomposite containing ZnO NPs and *F. gummosa* essential oil on the amount of TVB-N of fish fillets during 16 days of storage at refrigerator temperature is shown in Fig. 6. The amount of TVB-N on day 0 for all samples was measured around 12.30 mg per 100 g of fish fillet, which was consistent with the results of Chamanara et al.^39^. According to the results, the amount of TVB-N index increased from the 0 day to the end of the storage period, and from the eighth day of storage to the end of the storage period, there was a significant difference between the control and the coated samples (*P* < 0.05). According to the standard, the maximum acceptable level of volatile nitrogen bases in aquatic meat is 35 mg of nitrogen per 100 g of meat^47^. In this study, on the sixth day, the control reached 47.92 ± 1.8 mg per 100 g of sample, which exceeded the permitted standard range and became unusable, but the other samples remained within the permitted range and continued until the end of the period. They were kept within the allowed range. The results of this study showed that starch nanocomposite and *F. gummosa* essential oil were effective in inhibiting enzymes and microbial activities. The delay in the formation of TVB-N in the coated samples can be due to the delay in the increase of the population of pathogenic bacteria and ultimately the reduction of the capacity of these bacteria for the oxidative deamination of non-protein nitrogenous compounds^48^. The antimicrobial activity can be attributed to the antimicrobial properties of *F. gummosa* essential oil. In the study of Khezari Ahmedabad et al.^49^, the effect of whey protein coating on the microbial quality of rainbow trout fillets was investigated and it was reported that the amount of volatile nitrogenous bases in the samples increased with increasing storage time and at the end of storage the coated samples containing 1.5% thyme essential oil had less volatile nitrogen bases than other samples. Chamanara et al.^39^ reported in the study of rainbow trout that chitosan film with thyme essential oil was effective in delaying the formation of volatile nitrogen bases.

### Microbial analysis of coated fish fillets

#### Total bacterial count

**Figure 7:**
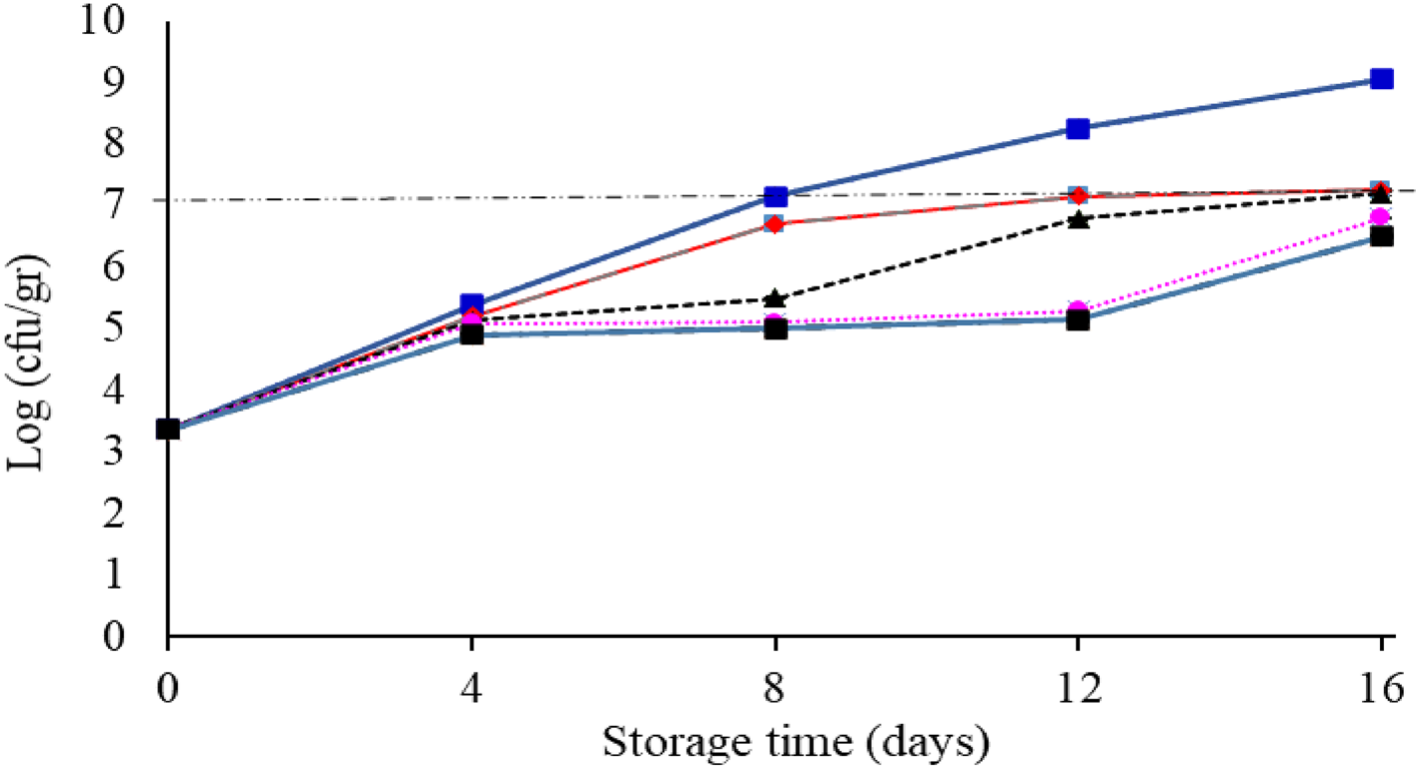
Changes in the mean (± standard deviation) of the total bacterial count of salmon fillet during 16 days of storage at refrigerator temperature.

The change in the total bacterial count of fish fillets in the control and the coated group is shown in Fig. 7. The initial number of bacteria was counted around 3.36 log CFU/g, which is almost consistent with the results of Nowzari et al.^50^. According to the results during the storage period, the total bacteria count had an increasing trend. From the eighth day of storage to the end of storage period, a significant difference (*P* < 0.05) was observed between the tested samples. The highest total count increase was observed in the control on day 16 with 9.05 log CFU/g and the lowest for the starch + 1.5% *F. gummosa* essential oil with 6.5 log CFU/g. The decrease in the growth rate of bacteria in the group of starch and essential oil nanocomposite is due to the antimicrobial effect of *F. gummosa* essential oil, which has a synergistic effect with the starch coating. Ojaq et al.^51^ reported that the chitosan coating and cinnamon essential oil significantly reduced the amount of final aerobic mesophilic bacteria compared to the control. Volpe et al.^52^ also investigated the effect of carrageenan coating with lemon extract on salmon and reported that from the 6th day onwards, a significant difference was observed between the number of mesophilic bacteria in the control and coated groups**.**

Heydari et al.^53^ investigated the effect of Qodume Shirazi (*Alyssum homolocarpum*) seed mucilage and lavender essential oil on the preservation of ostrich meat. From a microbiological point of view, the cold storage duration for the control and the coated sample without the essential oil was only 3 days, while for coated samples containing 0.5%, 1%, 1.5%, and 2% essential oil, it was 3, 3, 6, and 9 days, respectively. The coated ostrich meat containing 2% essential oil had an appropriate quality with an expanded shelf life.

#### Psychrophilic bacteria

**Figure 8:**
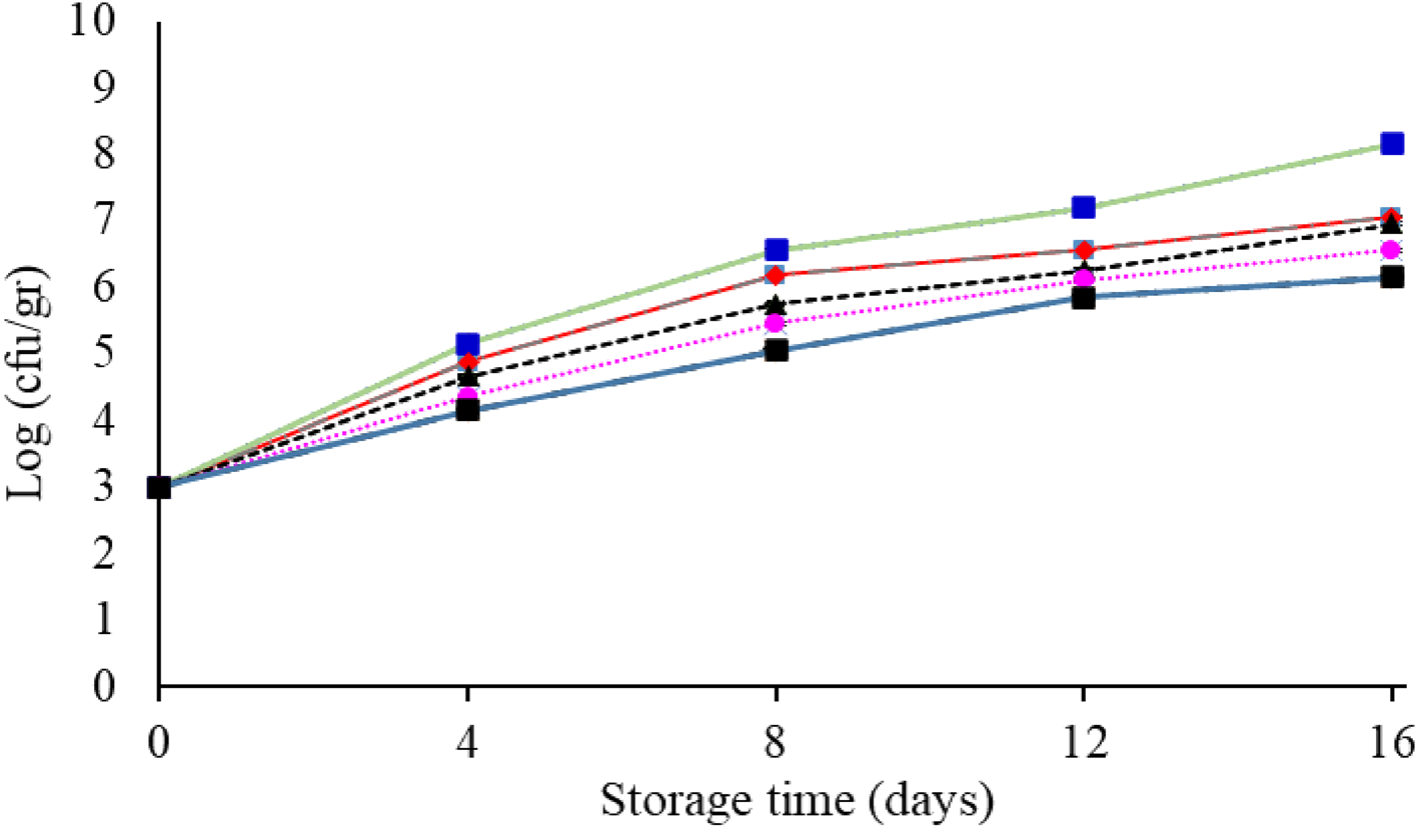
Changes in the mean (± standard deviation) of the psychrophilic bacteria of salmon fillet during 16 days of storage at refrigerator temperature.

The count of psychrophilic bacteria in the samples of the control and coated groups is shown in Fig. 8. The initial count of psychrophilic bacteria was 0.3 log CFU/g, which is consistent with the results of Ojagh et al.^51^. An increase in the number of psychrophilic bacteria with the passage of time was observed in all samples. From the fourth day of storage to the end of the storage period, a significant difference was observed between the control and the coated samples (*P* < 0.05). The lowest amount of psychrophilic bacteria was observed in the starch film + 1.5% of *F. gummosa* essential oil. In the study of Nasiri et al.^54^ on the effects of the aqueous extract of the *Myrtus communis* leaf on the bacterial changes of rainbow trout, kept at refrigerator temperature, the results showed that the immersion of the fish in the extract reduced the psychrophilic bacterial count. The average number of psychrophilic bacteria gradually increased until the end of the period. So that the number of psychrophilic bacteria in the control samples reached 8.15 log CFU/g on the 16th day. In samples treated with essential oil, this increase showed a slower trend. In a study conducted by Ojaq et al.^51^ on rainbow salmon, it was reported that chitosan coating and cinnamon essential oil significantly reduced the amount of psychrophilic bacteria during the storage period compared to the control.

Alghooneh et al.^55^ evaluated the effect of *Satureja extracts* (water and ethanol) on the population dynamics of *Pseudomonas aeruginosa* in Frankfurter sausage and reported that Satureja bachtiarica ethanol extract was able to reduce *P. aeruginosa* population, showing stronger effect at 5 °C and the concentration of 8000 ppm.

In a study the effect of *Plantago major* seed mucilage and Citrus Limon essential oil as edible coating on shelf-life of buffalo meat was investigated. The edible coating was able to significantly reduce the microbial growth (total viable count*, psychrotrophic* bacteria*, E. coli, S. aureus*, and fungi) in buffalo meat during storage period in comparison with the control^44^.

#### Lactic acid bacteria

**Figure 9:**
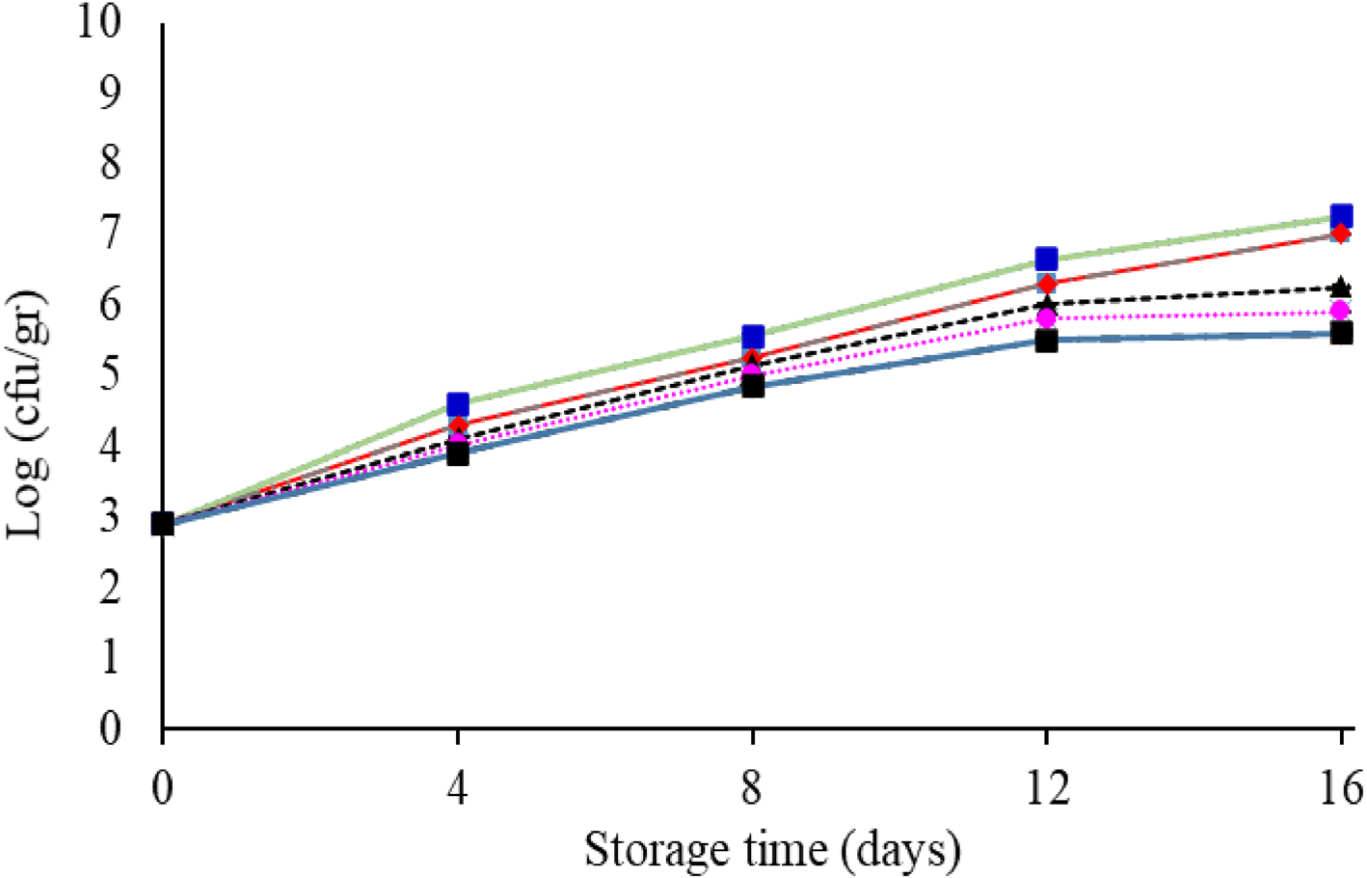
Changes in the mean (± standard deviation) of the lactic acid bacteria of salmon fillet during 16 days of storage at refrigerator temperature.

According to Fig. 9, it can be seen that lactic acid bacteria had an increasing trend during the storage period. The highest number of lactic acid bacteria was counted on day 16 for the control sample 7.25 log CFU/g. This effect was also observed in Volpe et al.^52^ studies. They reported that lactic acid bacteria in the samples containing carrageenan and carrageenan with lemon extract had a lower growth rate than the control. Kostaki et al.^43^ reported that lactic acid bacteria are among the most resistant Gram-positive bacteria to antimicrobial activity, but their growth was delayed by using coating with modified packaging and thyme essential oil.

## Conclusion

The study demonstrated that the thickness of the production coating was increased by the simultaneous addition of nanoparticles and essential oil. Similarly, the permeability to water vapor was improved when essential oil and nanoparticle were added together. Furthermore, the mechanical properties of starch were enhanced by the addition of ZnO NPs. The produced coating exhibited excellent antimicrobial and antioxidant activity, which could be attributed to the presence of active phenolic compounds such as alpha and beta pinene in the thyme essential oil. These compounds have been proven effective in previous studies.

## Data Availability

All the data generated/ analyzed during the study are available with the corresponding author on reasonable request.
